# Expanding HTA – Correcting a Misattribution, Clarifying the Scope of HTA and CEA: Comment on "Ethics in HTA: Examining the ‘Need for Expansion’"

**DOI:** 10.15171/ijhpm.2019.73

**Published:** 2019-09-16

**Authors:** Anthony J. Culyer

**Affiliations:** Department of Economics and Related Studies and Centre for Health Economics, University of York, York, UK.

**Keywords:** HTA, Cost-Effectiveness Analysis, Scope, Social Value Judgments

## Abstract

Abrishami, Oortwijn, and Hofman (AOH) attribute to me a position I do not hold and an argument I did not make. The purpose of this note is make clear what my position actually is and to clarify the main differences between health technology assessment (HTA) and cost-effectiveness analysis (CEA).

## A Misattribution

While I find myself in agreement with a good deal of what they write (for example, that health technology assessment [HTA] should be user-centred and suitable for legitimising decisions), Abrishami, Oortwijn, and Hofman (henceforth AOH) attribute^1^ to me a position I do not hold and an argument I did not make.^2^ This commentary aims to clarify a misattribution made by AOH. In a discussion of the editorial by Daniels and colleagues^3^ they claim that I make an “underlying,” and therefore implicit, assumption to the effect that HTA has a pre-existing “framework” with specific disciplinary associations that is “widely agreed.” Apparently, my view is also that this framework has “boundaries.” It is the boundaries that define the possible need for “expansion” so as to include matters beyond them.

## Is the Scope of an HTA Determined a Priori?

This is not my view and, in my opinion, is not a particularly helpful way of determining the scope of HTA. My view is that the proper scope of HTA is determined by the problem it is being harnessed to elucidate, which in turn is determined by the “ownership” of that problem (ie, the decision-makers in question) and the circumstances or context in which the decision is to be made. These circumstances include the country, its history and culture, the resources available, the political climate and the implementability of any actions decided upon after analysis. The choice being considered in an HTA is bounded only by considerations such as these. It follows that it is not necessary for the HTA analyst to agonise about the “need for expansion.” The range of considerations will be determined by the social values chosen by decision-makers, or given to them by higher authorities.^4,5^ This range is contextual. The HTA analyst has the job of eliciting both the values to be respected in any analysis and judging the feasibility of various options, given the policy objectives of whomever is commissioning the work (typically a department of government or a senior manager in an insurance company). What is socially right and ethical is not, therefore, to be decided a priori but in the light of the specific context.

Many of the elements of an HTA are rich in terms of social value judgments: the budget available will depend upon priorities established between alternative uses for resources inside and outside healthcare; the measures of outcome (like lives saved, quality-adjusted life year and disability-adjusted life year) are deeply imbued with social values that give meaning to some concept of “health” and that may vary according to whose life is likely to be affected (like babies, children, adults, the elderly, the mentally ill, informal family carers, people with multiple morbidities); the likely consequences of a decision for the distribution of the burdens of sickness and of healthcare expenditures; the acceptability of degrees of risk under conditions of uncertainty. There are also other kinds of judgment that are often required: how good or complete the research evidence; what the balance should be between quantitative and qualitative evidence; how transferable the results obtained in one study in another are to the country in question; how competently the systematic reviews, research summaries and all other supporting analyses have been done; how acceptable the necessary changes are in affected persons’ political and financial interests; how willing the professional groups are who are essential to implementation.

In principle, any or all of these factors (or indeed others) can be elements of an HTA. Whether in any specific context each or them is relevant depends on the context.

AOH advance the social value judgment that “the needs of the population must be the prime criteria.” This sounds innocuous enough but, since the very purpose of an HTA is to help decide what it is that is needed and by whom, it is circular and uninformative. Moreover, the interest may not be in the needs as such but in their distribution in a population or subgroup of a population.

The very first task in any HTA is therefore to establish as precisely as one can what the question is.^5^ This normally entails identifying one or more interventions that can affect health for the better; settling what is meant by “health”; deciding the criteria for choosing between the interventions (cost-effectiveness, equity, sustainability, religious proscriptions); various speeds of implementation; identifying potential gainers and losers; evaluating what other services will necessarily be forgone as a consequence of a decision to spend; making interpersonal comparisons between ethical claims to benefit; deciding who will be consulted and otherwise involved in the decision-making process^6-8^; identifying any training needs required for conducting the analysis and for implementing its results; and conveying the recommendations to “board level” authorities, clinical professionals, managers, organised patient groups and the general public.

Cost-effectiveness analysis (CEA) is a part of HTA but ought not to be identified with it. CEA does indeed have specific disciplinary roots (notably relevant clinical disciplines, economics, epidemiology and bio-statistics, and information science) and has a specific criterion (ie, cost-effectiveness). HTA is usually broader. It embraces cost-effectiveness of course, along with its associated disciplines, but often also requires the insights of ethics, theories of justice, management science, political science as well as others needed because of the specific nature of the interventions under consideration.

## Need for Pragmatism

HTA can be a costly business. The scope and the procedures I have just described will not always be judged worthwhile. But whether the scope be broad and the procedures “legitimate,” or neither, is not determined by any “framework” or restrictive “framing.” Nor is it restricted to the practitioners of a conventional set of disciplines or professions. Regarding the former, the basic requirement is simply that an HTA address the issues considered to be significant by the decision-makers and their expert advisers. Regarding the latter, one requires only sufficient competence to deliver an analysis that meets conventional professional standards^9^ and that has political credibility.

## Ethical issues

Not applicable.

## Competing interests

Author declares that he has no competing interests.

## Author’s contribution

AJC is the single author of the paper.
